# Real-world experience of women using extended-cycle vs monthly-cycle combined oral contraception in the United States: the National Health and Wellness Survey

**DOI:** 10.1186/s12905-017-0508-6

**Published:** 2018-01-18

**Authors:** Rossella E. Nappi, Iñaki Lete, Lulu K. Lee, Natalia M. Flores, Marie-Christine Micheletti, Boxiong Tang

**Affiliations:** 1 Research Center for Reproductive Medicine, Gynecological Endocrinology and Menopause, IRCCS Policlinico San Matteo Foundation, Department of Clinical, Surgical, Diagnostic and Pediatric Sciences, University of Pavia, Piazzale Golgi 2, 27100 Pavia, Italy; 2 Araba University Hospital, Jose Atxotegi Street, Vitoria, Spain; 3 Kantar Health, 393 Vintage Park Drive, Suite 100, Foster City, CA USA; 4 Teva Europe Women’s Health, Piet Heinkade 107, Amsterdam, The Netherlands; 5 Teva Pharmaceuticals, 41 Moores Road, Frazer, PA USA

**Keywords:** Real-world, Extended-regimen, Heavy menstrual bleeding, Menstrual cycle, Satisfaction, Adherence, Women’s health, Hormonal contraception

## Abstract

**Background:**

The real-world experience of women receiving extended-cycle combined oral contraception (COC) versus monthly-cycle COC has not been reported.

**Methods:**

Data were from the United States 2013 National Health and Wellness Survey. Eligible women (18–50 years old, premenopausal, without hysterectomy) currently using extended-cycle COC (3 months between periods) were compared with women using monthly-cycle COC. Treatment satisfaction (1 “extremely dissatisfied” to 7 “extremely satisfied”), adherence (8-item Morisky Medication Adherence Scale^©^), menstrual cycle-related symptoms, health-related quality of life (HRQOL) and health state utilities (Medical Outcomes Short Form Survey-36v2®), depression (9-item Patient Health Questionnaire), sleep difficulties, Work Productivity and Activity Impairment-General Health, and healthcare resource use were assessed using one-way analyses of variance, chi-square tests, and generalized linear models (adjusted for covariates).

**Results:**

Participants included 260 (6.7%) women using extended-cycle and 3616 (93.3%) using monthly-cycle COC. Women using extended-cycle COC reported significantly higher treatment satisfaction (*P* = 0.001) and adherence (*P* = 0.04) and reduced heavy menstrual bleeding (*P* = 0.029). A non-significant tendency toward reduced menstrual pain (39.5% versus 47.3%) and menstrual cycle-related symptoms (40.0% versus 48.7%) was found in women using extended-cycle versus monthly-cycle COC. Significantly more women using extended-cycle COC reported health-related diagnoses, indicating preferential prescription for extended-cycle COC among women reporting more health problems. Consistent with this poorer health, more women using extended-cycle COC reported fatigue, headache, and activity impairment (*P* values < 0.05). There were no other significant differences between groups.

**Conclusions:**

This real-world observational study supports extended-cycle COC as a valuable treatment option with high satisfaction, high adherence, and reduced heavy menstrual bleeding.

## Background

Women in the United States (US) and Europe who use hormonal contraception most commonly select combined oral contraception (COC) [1]. Standard 21/7 COC, including 21 days of active pills followed by a 7-day hormone-free interval, was developed to induce monthly bleeds that mimic the natural menstrual cycle; however, no health benefits of induced monthly bleeding have been demonstrated [2]. In contrast, negative effects associated with monthly bleeding, including disruption of daily life due to menstrual cycle-related discomfort and/or inconvenience, have been shown [3, 4]. Additionally, surveys of women in the US and Europe have shown a majority report a preference for reduced frequency of menstrual bleeding to every 3 months or contraceptive-induced amenorrhea [4–7].

Extended-cycle COC regimens, which include >28 days of active pills and reduce scheduled bleeding episodes to 4 per year, are effective in pregnancy prevention and their safety profile is similar to 28-day cyclical regimens [8, 9]. Due to the reduced frequency of scheduled bleeding compared with monthly-cycle COC, extended-cycle COC may provide greater improvement in menstrual cycle-related medical conditions and symptoms, such as dysmenorrhea, premenstrual syndrome (PMS), menstrually related headaches, endometriosis, heavy menstrual bleeding, iron deficiency, and anemia [10, 11]. Additionally, extended-cycle COC may reduce menstrual cycle-related inconvenience and interference with daily activities, work/school attendance, personal social events, and sports. Altogether, these positive benefits of extended-cycle COC may lead to greater treatment satisfaction and adherence, improved health-related quality of life (HRQOL), and economic benefits related to reduced expenses for feminine-hygiene products, pain medication, and medical consultations [10, 11].

Extended-cycle COC has been available in the US since 2003 [12], with more recent availability in Europe; however, the real-world experience of women receiving extended-cycle COC has not yet been described. The current study examined the real-world experience of women in the US receiving extended-cycle COC versus monthly-cycle COC and characterized treatment satisfaction, adherence, and menstrual cycle-related symptoms, as well as HRQOL, health-related work and activity impairment, and healthcare resource use.

## Methods

### Study design

Study data were from the 2013 National Health and Wellness Survey (NHWS), a nationally representative, self-administered, internet-based survey of adults. The NHWS is a large scale general population survey in the healthcare industry. The annual survey is collected in the US, Europe, Japan, China, Brazil, and Russia. NHWS respondents are recruited from an internet panel using a random stratified sampling framework to ensure the demographic composition (ie, age, gender, and ethnicity for the US respondents) is representative of the adult population of the country. The current study examined the US data from the 2013 NHWS. Electronic consent was obtained from all participants in the NHWS. The survey was granted exemption by the Essex Institutional Review Board (Lebanon, NJ).

Eligible women met the study inclusion criteria of 18 to 50 years old, premenopausal, without hysterectomy, and self-reported current use of COC. Women who reported using extended-cycle COC with 3 months between periods were compared with women who reported using monthly-cycle COC with 3 to 4 weeks between periods.

### Assessments

Demographic and health characteristics were reported, including comorbid health-related diagnoses and comorbid disease burden using the Charlson Comorbidity Index (CCI) [13]. The weighted presence of 18 conditions is used to create a CCI score that ranges from 0 to 37, with higher scores indicating greater comorbid disease burden [14].

Primary treatment-related outcomes included satisfaction with current COC, adherence to the COC regimen, and menstrual cycle-related symptoms. Treatment satisfaction was rated from 1 (“extremely dissatisfied”) to 7 (“extremely satisfied”). Adherence was assessed using the 8-item Morisky Medication Adherence Scale^©^ (MMAS-8; used under license and with permission) [15], a validated self-report measure of adherence to prescribed medications. Lower scores represent lower adherence (range from 0 to 8). Assessment of menstrual cycle-related symptoms included the presence in the past month of heavy menstrual bleeding, menstrual pain, dysmenorrhea, and 15 symptoms experienced as a result of PMS/premenstrual dysphoric disorder (PMDD).

Secondary health outcomes included HRQOL, depression, and sleep difficulties, and economic outcomes examined work productivity and activity impairment and healthcare resource use. HRQOL was assessed using the physical component summary (PCS) and mental component summary (MCS) scores from the Medical Outcomes Study 36-item Short Form Survey version 2® (SF-36v2) [16]. PCS and MCS scores are normed to a mean of 50 ± 10, with higher scores indicating better health status. The SF-36v2 was also used to generate the health state utilities Short Form-6 Dimensions (SF-6D) index score, using the United Kingdom general population health state preference weights [17]. The SF-6D utilities index scale anchors range from 0 (health equivalent to death) to 1 (health equivalent to full health) and calculated index scores range from 0.29 to 1.0 [18]. Higher scores indicate better health status; the general population mean is 0.78 [19]. Depression was examined using the 9-item Patient Health Questionnaire (PHQ)-9 [20]. Depression severity was assessed as no to minimal depression (score 0 to 4), mild (score 5 to 9), moderate (score 10 to 14), moderately severe (score 15 to 19), and severe (score 20 to 27). Sleep difficulties were assessed as the presence of 12 sleep-related problems.

The Work Productivity and Activity Impairment-General Health (WPAI-GH) questionnaire, a 6-item validated assessment [21], examined outcomes due to one’s health in the past 7 days. Absenteeism (percentage of work time missed due to one’s health problems), presenteeism (percentage of impairment experienced while at work due to one’s health problems), overall work impairment (overall work productivity loss calculated from the combined absenteeism and presenteeism scores) and activity impairment (percentage of impairment in daily activities due to one’s health problems) were examined. Women who were full-time or part-time employed provided responses for absenteeism, presenteeism, and overall work impairment. All women provided a response for activity impairment. Higher percentages indicate greater impairment and less productivity. Healthcare resource use (past 6 months, all-cause) included the number of any traditional healthcare provider visits, general practitioner visits, emergency room visits, and hospitalizations.

### Data analysis

Unadjusted one-way analysis of variance (ANOVA) or chi-square tests were completed for demographic characteristics and all outcomes. Generalized linear models that adjusted for covariates were used to examine treatment satisfaction, adherence, heavy menstrual bleeding, and menstrual cycle pain. Covariates included demographics (age, insurance coverage of contraception), health characteristics (body mass index [BMI]), and comorbidities (CCI, migraine/headaches, depression, anxiety, and sleep difficulties). Adjusted means with 95% confidence intervals (CIs) are reported. Analyses were completed using SPSS version 23.0 (Chicago, IL) and *P* < 0.05 (2-tailed) was considered statistically significant.

## Results

### Participant characteristics

The 2013 NHWS US sample included 75,000 participants. Eligible women included in the current study totaled 3876. Use of extended-cycle COC was reported by 6.7% (260/3876) of women and monthly-cycle COC by 93.3% (3616/3876). Women using monthly-cycle COC reported longer duration of COC use (58.9 ± 62.8 months) compared with women using extended-cycle COC (46.8 ± 36.8 months; *P* = 0.002).

Women using extended-cycle COC were slightly older (31.6 years vs 30.3 years, *P* = 0.011) and more frequently reported health insurance coverage of contraception (78.5% vs 70.9%, *P* = 0.01; Table 1). There was no significant difference between women in the extended-cycle and monthly-cycle COC groups in mean CCI score; however, a significantly greater percentage of women receiving extended-cycle COC reported diagnoses of migraines (27.3% vs 15.9%), headaches (21.2% vs 15.7%), sleep difficulties (15.8% vs 10.1%), heartburn (15.8% vs 10.8%), hypertension (10.0% vs 6.3%), and irritable bowel syndrome (9.6% vs 5.8%), indicating preferential prescription of extended-cycle COC among women with greater health problems.

**Table 1:** Demographic characteristics of women using extended-cycle or monthly-cycle COC

	Extended-cycle COC n = 260	Monthly-cycle COC n = 3616
Age, mean ± SD^a^	31.6 ± 7.7	30.3 ± 7.6
CCI, mean ± SD	0.1 ± 0.4	0.1 ± 0.5
Race/Ethnicity, n (%)		
Non-Hispanic White	205 (78.8)	2720 (75.2)
Non-Hispanic Black	18 (6.9)	303 (8.4)
Hispanic	17 (6.5)	296 (8.2)
Other ethnicity	20 (7.7)	297 (8.2)
Education, n (%)		
< 4-year college degree	107 (41.2)	1583 (43.8)
≥ 4-year college degree	153 (58.8)	2033 (56.2)
BMI, n (%)^a^		
Underweight	7 (2.7)	150 (4.1)
Normal weight	141 (54.2)	1822 (50.4)
Overweight	42 (16.2)	838 (23.2)
Obese	61 (23.5)	723 (20.0)
Unknown	9 (3.5)	83 (2.3)
Marital status, n (%)		
Single/divorced/separated/widowed	127 (48.8)	1737 (48.0)
Married/living with partner	133 (51.2)	1879 (52.0)
Have health insurance, n (%) Yes	237 (91.2)	3199 (88.5)
Health insurance covers COC, n (%) Yes^a^	204 (78.5)	2565 (70.9)
Current smoker, n (%) Yes	22 (8.5)	362 (10.0)
Exercise ≥ 20 min ≥ 1 time past month, n (%) Yes	207 (79.6)	2827 (78.2)

BMI, body mass index; CCI, Charlson Comorbidity Index; COC, combined oral contraception

^a^*P* < 0.05

### Satisfaction, adherence, and menstrual cycle symptoms

Women using extended-cycle COC reported significantly higher treatment satisfaction (adjusted mean 6.1 [95% CI: 5.9, 6.2]) versus women on monthly-cycle COC (adjusted mean 5.8 [95% CI: 5.8, 5.9], *P* = 0.001; Table 2). Women using extended-cycle COC reported significantly greater adherence (adjusted mean 6.9 [95% CI: 6.7, 7.1]) versus women using monthly-cycle COC (adjusted mean 6.7 [95% CI: 6.7, 6.8], *P* = 0.04).

**Table 2:** Treatment satisfaction, heavy menstrual bleeding, and menstrual pain

	Extended-cycle COC	Monthly-cycle COC
	Adjusted Mean (95% CI)	Adjusted Mean (95% CI)
Treatment satisfaction^a^	6.1 (5.9, 6.2)	5.8 (5.8, 5.9)
Treatment adherence (MMAS-8)^a^	6.9 (6.7, 7.1)	6.7 (6.7, 6.8)
Heavy menstrual bleeding (% women)^a^	8.6 (5.8, 12.4)	13.0 (11.9, 14.2)
Menstrual pain in past month (% women)	39.5 (29.1, 50.9)	47.3 (44.2, 50.5)

CI, confidence interval; COC, combined oral contraception; MMAS-8, Morisky Medication Adherence Scale

^a^*P* values < 0.05 in generalized linear models adjusted for covariates

Significantly fewer women using extended-cycle COC reported heavy menstrual bleeding (adjusted mean percentage 8.6% [95% CI: 5.8%, 12.4%] vs monthly-cycle COC 13.0% [95% CI: 11.9%, 14.2%], *P* = 0.029; Table 2). Fewer women receiving extended-cycle COC appeared to report menstrual pain in the past month (adjusted mean percentage 39.5% [95% CI: 29.1%, 50.9%]) versus monthly-cycle COC (47.3% [95% CI: 44.2%, 50.5%]); however, the difference was not statistically significant (Table 2).

**Fig. 1:**
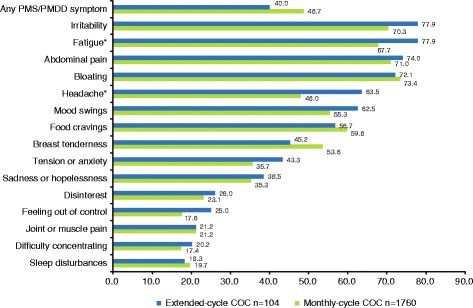
Percentage of women reporting PMS/PMDD symptoms COC, combined oral contraception; PMS/PMDD, premenstrual syndrome/premenstrual dysphoric disorder. Percentage of women reporting any PMS/PMDD symptom includes all extended-cycle (*n* = 260) and monthly-cycle (*n* = 3616) women. Percentage of women reporting specific PMS/PMDD symptoms includes only those women who reported any PMS/PMDD symptom (extended-cycle, *n* = 104; monthly-cycle, *n* = 1760). *P < 0.05, chi-square tests. Symptoms of fatigue and headache were reported by significantly more women receiving extended-cycle versus monthly-cycle COC

PMS/PMDD symptoms were reported by 40.0% (104/260) of women using extended-cycle COC and 48.7% (1760/3616) of women using monthly-cycle COC (Fig. 1). Among these women, the most frequently reported symptoms were abdominal pain (74.0% of women using extended-cycle COC; 71.0% of women using monthly-cycle COC), bloating/fluid retention (72.1%; 73.4%), fatigue (77.9%; 67.7%), and irritability (77.9%; 70.3%). Fatigue and headache were reported by a significantly greater percentage of women using extended-cycle COC versus monthly-cycle COC (*P* values < 0.05).

### HRQOL, depression, and sleep difficulties

**Fig. 2:**
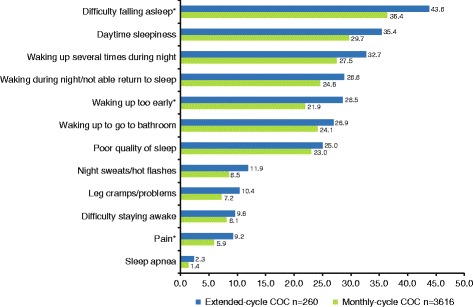
Percentage of women reporting sleep difficulties COC, combined oral contraception. *P < 0.05, chi-square tests. Women reported the presence versus absence of 12 sleep difficulties. Difficulty falling asleep, sleep difficulty associated with pain, and waking up too early were reported by significantly more women receiving extended-cycle COC compared with women receiving monthly-cycle COC

There were no significant differences between women using extended-cycle or monthly-cycle COC in PCS (unadjusted mean 54.4 ± 7.3 vs 54.4 ± 7.0, respectively), MCS (unadjusted mean 46.0 ± 11.1 vs 46.7 ± 10.2, respectively), or the SF-6D health utilities index score (unadjusted mean 0.7 ± 0.1 vs 0.8 ± 0.1, respectively) (Table 3). Depression did not significantly differ between women using extended-cycle (unadjusted mean 5.2 ± 5.5) or monthly-cycle (unadjusted mean 4.8 ± 5.3) COC and most women experienced no depression to mild depression (Table 3). Among the 12 assessed sleep difficulties (Fig. 2), significantly more women receiving extended-cycle COC versus women using monthly-cycle COC reported difficulty falling asleep (43.8% vs 36.4%, *P* = 0.016), pain (9.2% vs 5.9%, *P* = 0.03), and waking up too early (28.5% vs 21.9%, *P* = 0.014).

**Table 3:** HRQOL, health state utilities, depression, and healthcare resource use

	Extended-cycle COC	Monthly-cycle COC
	Mean ± SD	Mean ± SD
HRQOL		
Physical Component Summary	54.4 ± 7.3	54.4 ± 7.0
Mental Component Summary	46.0 ± 11.1	46.7 ± 10.2
Health state utilities		
SF-6D index score	0.7 ± 0.1	0.8 ± 0.1
Depression		
PHQ-9 score	5.2 ± 5.5	4.8 ± 5.3
Healthcare Resource Use		
Any traditional healthcare provider visits	4.3 ± 6.6	3.7 ± 5.9
General practitioner visits	1.1 ± 1.6	0.9 ± 1.8
Emergency room visits	0.2 ± 0.5	0.2 ± 1.2
Hospitalizations	0.1 ± 0.3	0.1 ± 0.4

COC, combined oral contraception; HRQOL, health related quality of life; PHQ-9, Patient Health Questionnaire-9; SD, standard deviation; SF-6D, Short Form-6 Dimensions

All *P* values > 0.05, unadjusted one-way ANOVA analyses

### Work and activity impairment and healthcare resource use

**Fig. 3:**
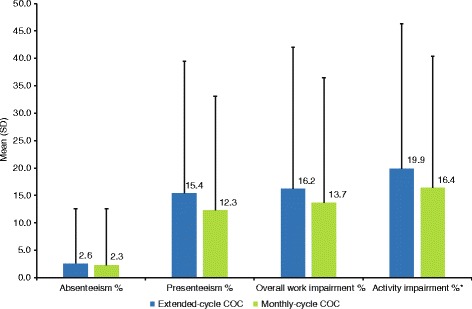
Work productivity and activity impairment due to health in the past 7 days COC, combined oral contraception; SD, standard deviation; WPAI-GH, Work Productivity and Activity Impairment-General Health. *P < 0.05, unadjusted one-way ANOVA analyses. Work productivity and activity impairment were assessed using the WPAI-GH. Women who were employed full or part-time (*n* = 173 extended-cycle COC; *n* = 2504 monthly-cycle COC) responded to work productivity questions (with the exception that *n* = 2493 women receiving monthly-cycle COC responded to the presenteeism question). All women responded to activity impairment questions. Women receiving extended-cycle COC reported a significantly greater mean percentage of impairment due to health in their daily activities over the past 7 days compared with women receiving monthly-cycle COC

Examination of work productivity and activity impairment due to health (Fig. 3) found no significant differences between women using extended-cycle or monthly-cycle COC in percentage of work time missed (absenteeism unadjusted means 2.6 ± 10.0% vs 2.3 ± 10.3%), percentage of impairment experienced at work (presenteeism unadjusted means 15.4 ± 24.1% vs 12.3 ± 20.8%), or overall work productivity loss (overall work impairment unadjusted means 16.2 ± 25.8% vs 13.7 ± 22.8%). Women using extended-cycle versus monthly-cycle COC reported a higher percentage of impairment in daily activities due to health (unadjusted mean 19.9 ± 26.4% vs 16.4 ± 24.0%, *P* = 0.025). Healthcare resource use (Table 3) did not significantly differ between women receiving extended-cycle versus monthly-cycle COC for the total number of visits to any traditional healthcare provider (unadjusted means 4.3 ± 6.6 vs 3.7 ± 5.9), general practitioner visits (unadjusted means 1.1 ± 1.6 vs 0.9 ± 1.8), emergency room visits (unadjusted means 0.2 ± 0.5 vs 0.2 ± 1.2), or hospitalizations (unadjusted means 0.1 ± 0.3 vs 0.1 ± 0.4).

## Discussion

COC has evolved to include extended-cycle COC regimens with 4 scheduled bleeding episodes per year. Many women in the US and Europe prefer less frequent menstrual cycles [4–7], and acceptance of extended-cycle COC continues to increase [8, 9, 12]. In line with reduced menstrual cycle-related symptoms in women using extended-cycle hormonal contraception [10, 11], common reasons for prescribing extended-cycle COC include relief from menstrual symptoms and convenience of menstrual suppression [12, 22–24].

The availability of the first 21/7 COC in 1960 revolutionized reproductive choice for women [25]. The development of extended-cycle COC allows women to reduce the discomfort and/or inconvenience associated with monthly menses, resulting in fewer menstrual cycle-related disruptions in social, sexual, and sport activities, concerns previously expressed by women [3–5]. Potential negative personal and societal economic effects include reduced academic/work force participation and increased costs associated with management of menstrual cycle disorders and symptoms and related healthcare resource use. Extended-cycle regimens significantly reduce use of menstrual-hygiene products and drug products to treat menstrual symptoms and may produce cost savings related to reduced work/school absenteeism and reduced doctor visits [26]. The potential benefit of extended-cycle regimens on sexual behavior and satisfaction requires further study but improvement has been suggested with a 72/4 COC regimen [27].

The current study examined the real-world experience of US women using extended-cycle versus monthly-cycle COC within the NHWS. Key findings included significantly higher treatment satisfaction and adherence and reduced heavy menstrual bleeding in women using extended-cycle COC. Additionally, fewer women using extended-cycle COC appeared to report menstrual pain in the past month or PMS/PMDD symptoms. The lack of statistical significance in these outcomes may be related to the finding of preferential prescription of extended-cycle COC in women reporting health problems. There was a greater frequency of health-related diagnoses reported by women using extended-cycle COC, including diagnoses for headaches, sleep difficulties, and health problems, and significantly more women using extended-cycle COC reported fatigue, headaches, sleep difficulties, and daily activity impairment due to general health. Poorer health among women using extended-cycle COC may have obscured group differences in non-contraceptive benefits, such as in well-being, work productivity, and healthcare resource use. In this real-world study, it is possible that women using extended-cycle COC were prescribed their COC, at least in part, because they already had worse functioning in these areas, possibly related to or exacerbated by menses. This possibility, and potential improvements in these outcomes following initiation of extended-cycle COC, could not be evaluated in the current study because the cross-sectional survey design does not allow an analysis of change from pre- (ie, no COC or monthly-cycle COC) to post-initiation of extended-cycle COC. Similarly, whether the poorer health in women using extended-cycle COC began prior to or following initiation of extended-cycle COC is not known. Prospective, controlled studies are needed to address these questions.

Study strengths include real-world data, drawn from the NHWS, which depict the real-life experience of women using extended-cycle COC and complement clinical trials. A large number of women and a broad range of health-related and economic-related measures were included. In the examination of work productivity and activity impairment, future studies may consider modifying the WPAI-GH 7-day reporting period and/or the focus of the health disruption to menstrual cycle-related, as has been done in women with endometriosis [28, 29] and heavy menstrual bleeding [30, 31]. Study limitations include the cross-sectional survey design, the consequent lack of information about menstrual- and health-related symptoms prior to using extended-cycle COC, and the smaller sample of women reporting extended-cycle versus monthly-cycle COC. Additionally, different formulations of extended-cycle COC, such as those with and without hormone-free intervals, of monthly-cycle COC, and type of progestin were not evaluated. And, as a real-world observational analysis, a sample bias may exist.

Given the small percentage of women using extended-cycle COC in the current study, there is clearly a continued need for improved awareness of the availability and utility of extended-cycle COC [12]. Almost half of the women using monthly-cycle COC reported PMS/PMDD symptoms, indicating the potential benefit of switching to COC with reduced frequency of menses [10, 11]. Specifically, the presence of any menstrual symptoms has been associated with significantly lower HRQOL [32], suggesting a benefit of reduced frequency of menses. Contraceptive counselling should evaluate bleeding preferences, which may vary with cultural background, and determine the potential fit of extended-cycle COC [2, 5]. Counseling should include identifying any misconceptions, educating patients that monthly bleeding is not necessary and is not an indication of health when using COC, and improve patient understanding of the safety and potential non-contraceptive benefits of extended-cycle COC [2].

## Conclusions

Extended-cycle COC with reduced frequency of menstrual cycles may help women better manage discomfort, inconvenience, and disruption of daily activities associated with monthly menses. The real-world experience of women using extended-cycle COC supports high treatment satisfaction and adherence and reduced heavy menstrual bleeding. Preferential prescription of extended-cycle COC was found among women reporting health problems, and poorer health may have obscured significant differences in broader non-contraceptive benefits when compared with monthly-cycle COC users. Although there are some limitations, this analysis provides information regarding patient experiences in a real-world setting that are not available from clinical trials, which may be useful for health care providers and patients in clinical practice. Further research examining extended-cycle COC and patient-reported outcomes of satisfaction, menstrual symptoms, HRQOL, and the economic impact of improved management of menstrual cycle-related symptoms is warranted.

## Abbreviations

ANOVA: analysis of variance
BMI: body mass index
CCI: Charlson Comorbidity Index
CI: confidence interval
COC: combined oral contraception
HRQOL: health-related quality of life
MCS: mental component summary
MMAS: Morisky Medication Adherence Scale
NHWS: National Health and Wellness Survey
PCS: physical component summary
PHQ-9: 9-item Patient Health Questionnaire
PMDD: premenstrual dysphoric disorder
PMS: premenstrual syndrome
SF-36v2: 36-item Short Form Survey version 2
SF-6D: Short Form-6 Dimensions
US: United States
WPAI-GH: Work Productivity and Activity Impairment-General Health
